# COVID-19: Relative Risk of Non-Vaccinated to Vaccinated Individuals

**DOI:** 10.3390/diseases10040113

**Published:** 2022-11-23

**Authors:** Davide Barbieri, Geza Halasz, Elisabetta Bertellini, Arianna Gaspari, Gabriele Melegari

**Affiliations:** 1 Department of Biomedical Sciences and Surgical Specialties, University of Ferrara, Corso Ercole I d’Este 32, 44121 Ferrara, Italy; 2 Department of Cardiology, Gugliemo da Saliceto Hospital, 29121 Piacenza, Italy; 3 Department of Intensive Care, Azienda Ospedaliero Universitaria di Modena, 41121 Modena, Italy

**Keywords:** pandemic, Italy, relative risk, likelihood ratios, non-linearity

## Abstract

Following the outbreak of the COVID-19 pandemic, Italy has implemented an extensive vaccination campaign involving individuals above the age of 12, both sexes. The public opinion and the medical community alike questioned the usefulness and efficacy of the vaccines against SARS-CoV-2. The widespread opinion was that the vaccines protected individuals especially against serious conditions which could require intensive care and may lead to the death of the patient rather than against the possibility of infection. In order to quantify the effect of the vaccination campaign, we calculated the relative risks of non-vaccinated and vaccinated individuals for all possible outcomes of the disease: infection, hospitalization, admission to intensive care and death. Relative risk was assessed by means of likelihood ratios, the ratios of the probability of an outcome in non-vaccinated individuals to the probability of the same outcome in vaccinated individuals. Results support the hypothesis that vaccination has an extensive protective effect against both critical conditions and death. Nonetheless, the relative magnitude of the protection in vaccinated individuals compared to those non-vaccinated appears to be higher against the former outcome than the latter, for reasons which need to be investigated further.

## 1. Introduction

Italy was the first European country to be struck by SARS-CoV-2 infections in early 2022. The pandemic rapidly spread, saturating intensive care units (ICUs) and claiming the lives of many Italians, especially senior citizens [1,2]. Since the inception of the pandemic, on the 21st of December 2021 the total number of deceased whose main cause of death was SARS-CoV-2 was officially 135,931. Data are updated and uploaded daily on GitHub by the Department for Civil Protection of the Italian government and are available in text format from the official data repository [3].

As soon as vaccines became available, the Italian government implemented an extensive vaccination campaign targeting individuals above the age of 12. Priority was given to the highest age class (80 years old and older) since age was rightly considered to be one of the most important risk factors [4]. The case fatality rate for individuals younger than 12 was considered so low to justify their exclusion from the current vaccination campaign, at least momentarily. Other characteristics which were taken into consideration in order to prioritize vaccination were chronic conditions and a state of immune-suppression or immune-depression, which could increase the risk [5,6].

The vaccines chosen by the Italian government for the vaccination campaign were the following: Comirnaty (BNT162b2) by Pfizer/BioNTech, Spikevax (mRNA-1273) by Moderna, Vaxzevria (ChAdOx1-S) by AstraZeneca, and Janssen (Ad26.COV2.S) by Johnson & Johnson [7].

As the campaign proceeded, medical institutions, political authorities and the public at large started to demand available data and reliable information about vaccines efficacy in order to obtain the possibility to evaluate whether the campaign was successful. Updates on the ongoing campaign are available on the official Italian government website [8]. In particular, the medical community has questioned the extent to which vaccines have reduced the risk of hospital admissions and fatalities. A study performed in the UK showed a significant reduction in the relative risk of hospital admissions in people who received two doses of vaccine compared to those who received just one [9]. The underlying hypothesis is that vaccinations should diminish especially the risk of a critical or fatal condition, rather than that of infection. This effect would reduce the pressure on the national health service and on ICUs in particular, besides mortality. The purpose of this study was to test this hypothesis, quantifying the efficacy of vaccines in diminishing COVID-19-related risks (especially hospitalization and death) compared to the non-vaccinated population.

## 2. Materials and Methods

Absolute numbers of vaccinated (vax) and non-vaccinated (no_vax) people in the overall Italian population above the age of 12 (the age class for which vaccination was planned) were taken from the official bulletin published by the Italian National Health Institute (Istituto Superiore di Sanità, ISS) on the 24 December 2021 [10]. Individuals who did not receive any vaccination or who had not completed a vaccination cycle (receiving only one dose of the vaccines for which at least two were planned) were counted in the no_vax group. Those who completed the vaccination cycle (that is, those who received two doses, or one for the vaccine for which only one was required), with or without booster dose, were counted in the vax group. The numbers of no_vax and vax individuals were further considered in the last 30 days before data collection per medical condition or outcome: infection, hospital admission, ICU admission and death, as summarized in Table 1.

Given an eligible population above the age of 12 of 54,009,944 people, the no_vax group included 8,534,032 individuals, while the vax group amounted to 45,475,912 individuals since the beginning of the campaign until the 21 December 2022 (data were updated till 3 days before publishing the bulletin).

In order to estimate the risk associated with each outcome including death, given the vaccination status, likelihood ratios (LRs) were calculated between the no_vax and vax groups. LRs represent the proportion of individuals with an outcome O in the two groups, no_vax or vax, that is, per vaccination status of the observed population. LRs can be calculated from the rule of Bayes for conditional probability as follows:

$$\frac{P(O|no\_vax)}{P(O|vax)}=\frac{P\left(no\_vax|O\right)P\left(vax\right)}{P\left(vax|O\right)P\left(no\_vax\right)}$$

where P(O|no_vax) is the probability of outcome O among no_vax, P(O|vax) is the probability of O among vax, P(no_vax|O) is the rate of no_vax among individuals with outcome O, P(vax|O) is the rate of vax among individuals with outcome O, P(vax) is the proportion of vax in the population, and P(no_vax) is the proportion of no_vax. The higher LR, the higher the risk of getting outcome O for the no_vax group.

## 3. Results

**Figure 1:**
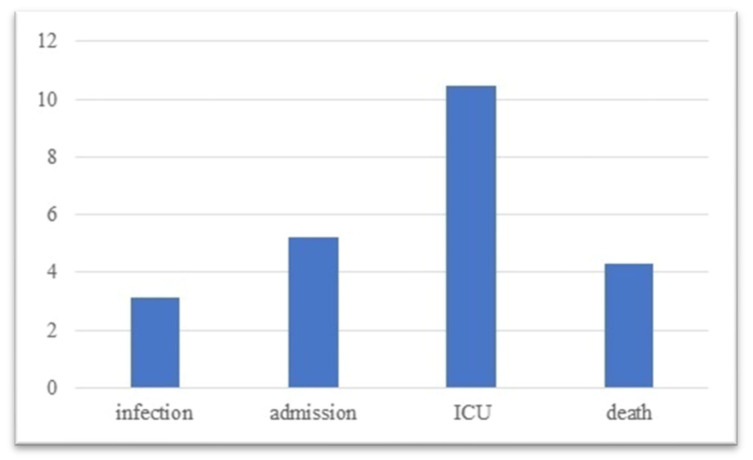
Relative risk distribution vs. outcome.

As of the 4th of December 2021, 15.8% of the target population was in the no_vax group, while the largest majority, 84.2%, was in the vax grpup. Among infected individuals 37% belonged to no_vax, while 63% belonged to vax, implying a 3.1 times higher risk for the no_vax individuals of becoming infected. Around half (49.5%) of hospital admissions were no_vax, the remaining were vax, implying a 5.1 times higher risk for no_vax individuals of being admitted to hospital. At the same time, 66.2% of ICU admissions were no_vax individuals, while the remaining 33.8% were vax, which implied a 10.4 times higher risk of becoming a critical patient for the former. Concerning fatalities, 44.7% were in the no_vax group, while 55.3% were in the vax group, which implied a 4.3 times higher risk for the former to die. Results are summarized in Table 2 and in Figure 1. As it can be seen in the chart, the risk for the no_vax group increased consistently as the condition worsened, with a notable exception when the outcome was death.

**Table 1:** No_vax and vax in the population above 12 and per medical condition.

Sample	No_Vax	Vax	Total
population	8,534,032	45,475,912	54,009,944
infected	149,746	254,999	404,745
admitted	6260	6384	12,644
ICUs	913	466	1379
deaths	893	1105	1998

## 4. Discussion

Estimating the efficacy of vaccinations in reducing the risks of SARS-CoV-2 infections—including their most serious consequences such as admission to intensive care and death—is not trivial and there is no general consensus on which index or proxy for vaccination efficacy should be preferred. Possible misunderstandings in the statistics behind them may partly explain this fact [11]. Further, most studies evaluated the reduction of attack rate, that is of infection, but not of the other outcomes as we did in our study.

The adoption of absolute risk reduction (ARR) has been suggested in order to avoid reporting or selection bias [12]. ARR can be defined as the difference between the risk of non-vaccinated individual to face a certain outcome and the risk of the vaccinated to face the same outcome, where the two risks are calculated as the percentages of individuals with a certain outcome in the no_vax and in the vax groups. Relative risk reduction (RRR) instead is preferred to ARR when the efficacy of a vaccine to diminish the risk of any outcome in case of a pandemic must be evaluated compared to a baseline risk of not being vaccinated [13,14]. RRR can be defined as 1-RR, where RR is the relative risk, i.e., the ratio of the risk of non-vaccinated to face an outcome and the risk of vaccinated to face the same outcome. It is correlated to ARR as it can also be calculated as ARR/(risk in the no_vax group).

Besides the fact that this is an observational study and not a randomized controlled trial which should be performed to calculate ARR and RRR, the main limitation of our study is the fact that only the outcomes of the last 30 days before data collection were available. In this case, the adoption of ARR was not consistent. In fact, the risk for any outcome in such a short time period is certainly underestimated for both groups, something which may affect absolute risk more than relative risk. In addition, we preferred to use LRs to estimate the relative risk of not receiving the vaccine because we considered them to be the most intuitive way to communicate our results in quantitative terms. Besides being derived from the rule of Bayes, LRs are correlated to RRR as LR = 1/(1 − RRR).

Since the percentage of no_vax individuals was significantly lower than that of vax individuals among the infected, the fact that the risk of being infected is higher for the no_vax group may appear counter-intuitive given the raw data. Still, this is consistent with the fact that the largest majority of the target population is vaccinated. Once the whole population will be eventually vaccinated, 100% of infected individuals will be in the vax group (possibly, very few). This perceived paradox may be explained as a consequence of a well-researched cognitive bias known as the base rate fallacy or base rate neglect [15]. The individual who evaluates the risk may in fact ignore the proportion of vax individuals in the population and consider only the distribution of no_vax and vax groups among infected individuals, getting the impression that the risk is higher for the latter. This is akin to assuming, consciously or not, the starting null hypothesis that the distribution of no_vax and vax groups in the population is even (the so-called equiprobability hypothesis [16]).

As expected instead, the relative risks of infection, hospital and ICU admission or death are all much higher in the no_vax group. This evidence supports the hypothesis of the efficacy of available vaccines in diminishing the risks associated with the infections caused by SARS-CoV-2, even if the relative risk of infection per se for the no_vax group is not as high as that of hospitalization or admission to intensive care. These results confirm the fact that vaccines play a major role in reducing the risk of a serious condition rather than that of becoming infected. Further, it must be considered that the relative risk is evaluated for the whole target population above the age of 12. If only older age categories, where the absolute risk is higher, were considered, vaccination would probably show a more significant effect [12]. Including individuals with and without booster dose in the same group may have diminished the magnitude of results, as booster doses may play an important role in reducing mortality even in the vaccinated population [17].

Our results are largely in agreement with the findings of Moghadas et al., who found a similar significant reduction of adverse outcomes in vaccinated individuals compared to non-vaccinated individuals in the USA [18]. Similarly, Marrone et al. found a reduced risk of severe cases in vaccinated compared to non-vaccinated individuals in some European countries, confirming old age as one of the main risk factors [19,20].

Nonetheless, in our study, there was an unexpected non-linearity in the distribution of relative risk, which consistently increased from infection to admission to ICU, but diminished from admission to ICU to death (even if it remained greater than 4 times in no_vax compared to vax). According to our hypothesis that risk should increase for no_vax individuals as the condition of the patients or the outcome became more adverse, we expected that in case of death the relative risk for no_vax individuals compared to vax individuals was greater than 10.4 (which is the relative risk for no_vax compared to vax individuals in case of admission to ICU). Interestingly enough, instead, the relative risk of death in the no_vax compared to the vax group is lower than that of hospital admission in the two groups, even if it is still higher than that of infection. This effect was not highlighted by other studies to the best of our knowledge.

A possible statistical explanation may be found in a non-uniform distribution of vaccines in the Italian population. There might have been, so far, a higher rate of vaccinations among fragile and/or older individuals. This assumption is supported by the data released by the Regional Health Service (Servizio Sanitario Regionale) of Emilia-Romagna, one of the regions in the north of Italy which was most affected by the pandemic. According to the report published on the 21 December 2021, 49.8% of the individuals in the age class above 60 years old were fully vaccinated, while only 25.5% in the 40–59-year-old age class had completed the vaccination cycle in that region [21]. We can certainly acknowledge age as a risk factor, even if this uneven distribution does not necessarily imply a lower propensity to vaccination among the youngest, since they were also the last to receive the vaccine, in chronological order.

Nonetheless, it cannot be ruled out that people who thought that they were at higher risk of serious consequences if infected by the virus (because of age or a medical condition) have decided to get vaccinated more promptly than other individuals, younger and without chronic illnesses, since vaccination was not mandatory. In any case, as patients became critical, the protection offered by the vaccine was less determinant, and individual characteristics—including age, chronic illnesses or immune conditions—could have played an important role in the outcome (be it death or recovery) of the treatment. Co-morbidities could have a stronger cause-and-effect relationship in case of death than in admission to ICUs. Of course, vaccinations against SARS-CoV-2 did not provide any protection against such chronic conditions.

Previous infections could play a role as well in explaining our findings, since they provided temporary immunization against SARS-CoV-2 [22]. This effect is present in both vaccinated and non-vaccinated individuals. However, if previously infected people were less prone to get vaccinated because they relied on acquired immunity, then this fact could have diminished the mortality risk in the no_vax group more than that of becoming critical, since we cannot assume that risk distribution for each different outcome is the same among vaccinated individuals and those non-vaccinated with acquired immunity from previous infections.

The lack of such information as the personal history of previous infections and of eventual chronical illnesses, as well as of other important demographic data such as age and sex, can be acknowledged as another limitation of our study. Further, there is no individual indication about which vaccines were used, as data were aggregated. Still, we cannot exclude the fact that different vaccines may imply different levels of protection against the outcomes of the disease.

## 5. Conclusions

In conclusion, even if the reported data and our findings support the hypothesis that vaccination effectively diminishes the risk for the infection to become serious, there is an interesting non-linearity when the outcome is death, and the relative risk is not as high as expected for the no_vax group compared to the vax group. Further research is therefore needed to fully understand the relative risk of death in case of infection by SARS-CoV-2.

## Figures and Tables

**Table 2:** Proportions and likelihood ratios of no_vax group compared to vax group per outcome.

Outcome	No_Vax	Vax	LR
Infections	37.0%	63.0%	3.1
Admissions	49.5%	50.5%	5.2
ICUs	66.2%	33.8%	10.4
Deaths	44.7%	55.3%	4.3

## Data Availability

Data on vaccinated (vax) and non-vaccinated (no_vax) people, infections, hospital admissions, ICUs and fatalities were obtained from the bulletin published by the Italian National Health Institute (Istituto Superiore di Sanità, ISS) on the 24 December 2021.
